# Is there an impact of surgeon's experience on in-hospital outcome in patients with operatively treated proximal humerus and humerus shaft fractures?

**DOI:** 10.1016/j.sipas.2024.100269

**Published:** 2024-12-19

**Authors:** Andrew Adams, Christina Lorenz, Valentin Neuhaus, Hans-Christoph Pape, Claudio Canal

**Affiliations:** a Division of Trauma Surgery, University Hospital Zurich (USZ), University of Zurich (UZH), Raemistrasse 100, 8091 Zuerich, Switzerland; b Department of Surgery, Cantonal Hospital Thurgau, Pfaffenholzstrasse 4, 8501 Frauenfeld, Switzerland

**Keywords:** Teaching, Proximal humerus and shaft fractures, Outcome

## Abstract

**Background:**

Proximal humerus and shaft fractures are common, comprising 10–11 % of all fractures. Progress in their management includes refined surgical techniques and implants, coupled with a deeper understanding of fracture patterns.

**Aims:**

This study examines the effect of surgical education on in-hospital outcomes for operatively treated proximal and humerus shaft fractures, aiming to enhance patient care and results.

**Material and Methods:**

This study analyzed cases from 1st of January 2010 until the 31st of December 2021 using data extracted from the Swiss working group for quality assurance in surgery, including patients with proximal humerus and shaft fractures who underwent surgical procedures like open reduction with internal fixation (ORIF), closed reduction with internal fixation (CRIF), external fixation, or prosthesis. Analysis included patient demographics, procedure details, and outcomes, comparing those with and without teaching of the surgical procedures. Binary logistic regression identified risk factors, with statistical significance set at *p* = 0.001.

**Results:**

A total of 6,654 patients were analyzed. Most were treated with ORIF (74 %) or CRIF (17 %). The average hospital stay was 6.5 days. Teaching surgeries, comprising 5.4 % of all procedures, were more common among patients with fewer comorbidities and with public insurance coverage. These surgeries took slightly longer to perform compared to non-teaching cases (120±65 min vs. 113±60 min, *p*= <0.001). Public insurance coverage, absence of comorbidities, and certain surgical procedures (CRIF and ORIF vs. prosthesis) were associated with surgery being a teaching case. Complications occurred in 8 % of patients, with no significant difference between teaching and non-teaching groups. Predictors of complications included higher American Society of Anesthesiologists-score, antibiotic use, anticoagulation therapy, fracture of shaft, higher age, and longer surgery duration.

**Conclusions:**

Educational status did not affect in-hospital mortality and morbidity in patients with a operatively treated shaft or proximal humeral fracture. However, teaching was an independent predictor of a prolonged duration of surgery. Despite the significant differences, the clinical outcome was comparable in both groups, therefore substantiating the advantages of teaching operations for both patient safety and resident education. They combine the competence of experienced surgeons with the training of residents, whilst ensuring the safety through oversight and best practices. Not only does this environment improve patient outcomes, but also provides residents with hands-on experience, thus helping them make critical decisions, building confidence and developing essential skills.

## Introduction

Proximal humerus and shaft fractures, accounting for roughly 10 % - 11 % of fractures encountered in clinical practice, have become increasingly recognized in recent years, particularly within the elderly population after low impact falls and in young adults after severe trauma, making them the third most prevalent fractures especially in older adults [1,2,3]. Thus, surgical intervention continues to gain recognition for its potential to accomplish optimal outcomes among the complex spectrum of different treatment options in their management [4]. The treatment of humerus fractures has improved over the past years with advancements in new implants, different surgical techniques and a more comprehensive insight of fracture patterns. Central to this evolution is the integral role of modern surgical education, encompassing deliberate intraoperative guidance and subsequent formative and summative feedback within the hospital environment [5]. These teaching operations often involve the participation of residents and junior consultants, who perform the operation under supervision. Furthermore, treatment of these humerus fractures largely depends on fracture morphology, patient factors, as well as on the preferred management by the surgeon [6]. To the best of our knowledge, there is scarce literature about the influence of teaching operations on in-hospital outcome in patients with proximal humerus and humeral shaft fractures.

The aim of this study is to examine and accentuate the influence of surgical education in the management of these fractures.

We strongly believe that understanding the role of teaching operations in this context ultimately leads to improved patient care, advanced education, and last but not least, better outcomes for patients suffering from these demanding fractures [7].

## Material and methods

### Study design

For this investigation, data was utilized from the Swiss working group for quality assurance in surgery (known in German as “Arbeitsgemeinschaft für Qualitätssicherung in der Chirurgie - AQC”) [8]. The AQC database encompasses surgical in-hospital cases from surgical departments throughout Switzerland from the past 29 years. Data for the AQC database is collected through a general documentation file and an operation-specific file.

Ethics committee exempted our study from obtaining specific consent from the local cantonal ethics committee due to the lack of identifiable patient information within the AQC database. The current investigation was conducted in accordance with the Strengthening the Reporting of Observational Studies in Epidemiology (STROBE) guidelines [9].

## Participants/ study subjects

We analyzed data between the time-period of the 1st of January 2010 until the 31st of December 2021, including all patients with proximal humeral fractures or shaft fractures (diagnostic codes S42.2 - S42.3 based on the World Health Organization's International Statistical Classification of Diseases and Related Health Problems (ICD-10)) [10]. Due to a very low sample size of teached distal humeral fracture operations, they were excluded from the analysis. Only those patients who have undergone a surgical procedure, such as open reduction with internal fixation (ORIF), closed reduction with internal fixation (CRIF), external fixation and prosthesis were included. Other procedures, namely closed reduction only or hardware removal (osteosynthetic material extraction (OSME)) were excluded. Moreover, the teaching status of the operation must have been reported, which was a voluntarily binary input box. In Switzerland, whether a case is performed as a teaching case generally depends on the responsible surgeon. Among 11′075 patients, 6′654 demonstrated a complete data set and met our inclusion criteria and were therefore selected for further analysis.

### Variables, outcome measures, data sources and bias

The data won from the AQC provided information on patient age, sex, American Society of Anesthesiologists score (ASA physical status classification system), type of admission (elective or emergency), insurance status (public or private), length of hospital stay, need of the intensive care unit (ICU) for longer than a day, comorbidities, diagnosis (S42.2 proximal humerus fractures, S42.3 humeral shaft fractures), and type of discharge (deceased, at home, rehabilitation clinic, nursing home, old people's home, and other). Moreover, information concerning the procedure were given: complications during hospital stay, duration of surgery, thromboembolism prophylaxis during hospital stay, antibiotics during hospital stay, the type of operation (ORIF, CRIF, external fixation and prosthesis), as well as the teaching status of the procedure. A teaching case refers to a surgical procedure with the intention of educating less experienced surgeons (usually residents). During such procedures more experienced surgeons (usually consultants) guide and instruct trainees, giving them the opportunity of hands-on experience and insights into different surgical techniques. Under the supervision of an experienced surgeon, upper arm fracture procedures are occasionally performed by residents or not yet so experienced junior consultants in Switzerland.

### Statistics

Data was extracted online from the AQC-database using the evaluation tool AdjumedAnalyze (Adjumed Services AG, Zurich, Switzerland) and further processed using the Statistical Package for Social Sciences (SPSS, Version 29, IBM Corp., Armonk, New York, United States).

Analysis between groups of categorical data was done using the Chi-squared test and presented as the number of patients and percentages. The student's *t*-test was used to assess differences in means between groups for numerical data.

Binary logistic regression was used to determine independent risk factors for teaching and for complications. A *p* = 0,001 was considered statistically significant, chosen to account for the large sample size and to minimize the risk of Type I errors.

## Results

### Cohort

Altogether 6654 patients with a mean age of 65 ± 18 years remained for analysis. In total, 66 % of the patients were female and 34 % male, 83 % of our studied patients had a proximal humerus facture and 17 % a shaft fracture. The fractures were most frequently treated with ORIF (74 %) and CRIF (17 %). External fixators (0.3 %) and prostheses (9 %) made up the final part of the performed procedures. Of all patients, 33 % had a private insurance, whilst the other 67 % were publicly insured. Most patients had an ASA score of I or II and most patients were admitted as an emergency (71 %), whereas the other 29 % had a planned admission. The average hospital stay of all patients lied at 6.5 ± 4.6 days (6.4 ± 4.5 days in the non-teaching group and 6.7 ± 5.1 days in the teaching group, *p* = 0.177).

### Operative treatment

In total, 5.4 % of all operations were carried out by residents in training, 33 % by junior consultants and the remaining 63 % by senior consultants. The ASA-score was similar in both groups. Patients who received a teaching intervention had fewer comorbidities (18 % vs. 23 % in non-teaching group) and demonstrated a greater prevalence of public insurance (83 % vs. 62 % in the non-teaching group) (Table 1).

**Table 1:** Teaching YES or NO; patient characteristics.

Parameter		Teaching NO (*n* = 5240)		Teaching YES (*n* = 1414)		p value
		**n**	**%**	**n**	**%**	
Age (years)	mean ± SD	65±18		64±19		0.714
Sex	female	3481	66	955	68	0.446
	male	1759	34	459	33	
ASA	I (healthy person)	1309	25	350	25	0.296
	II (mild systemic disease)	2815	54	735	52	
	III (severe systemic disease)	1075	21	318	23	
	IV (severe systemic disease that is a constant threat to life)	41	0.78	11	0.78	
Type of admission	registered, planned	1486	28	423	30	0.260
	emergency	3754	72	991	70	
Insurance	statutory	3265	62	1172	83	<0.001
	private	1975	38	242	17	
Length of stay in hospital (days)	mean ± SD	6.4 ± 4.5		6.7 ± 5.1		0.177
Length of stay preoperative (days)	mean ± SD	1.4 ± 1.9		1.5 ± 1.9		0.033
Length of stay postoperative (days)	mean ± SD	5.0 ± 3.7		5.2 ± 4.4		0.945
Need for intensive care (> 1 day)	yes	68	1.3	28	2.0	0.060
Comorbidity	yes	1217	23	260	18	<0.001
Complications during hospital stay	yes	406	7.7	124	8.8	0.223
Diagnosis	S42.2 Fracture of upper end of humerus	4326	83	1126	80	0.013
	S42.3 Fracture of shaft of humerus	914	17	288	20	
Discharge	deceased	17	0.32	6	0.42	0.005
	at home	3928	75	1040	74	
	rehabilitation clinic	424	8.1	109	7.7	
	nursing home	221	4.2	66	4.7	
	old people's home	218	4.2	38	2.7	
	other	432	8.2	155	11	

SD: Standard Deviation, ASA: American Society of Anesthesiologists classification system,.

Comparing the average duration of surgery, the teaching group was statistically significantly longer than the non-teaching group (120 ± 65 min vs. 113 ± 60, *p* < 0.001) (Table 2).

**Table 2:** Teaching YES or NO; Procedure characteristics.

Parameter		Teaching NO (*n* = 5240)		Teaching YES (*n* = 1414)		p value
		**n**	**%**	**n**	**%**	
Surgeon class	senior consultant	3899	74.4	277	19.6	<0.001
	junior consultant	1243	23.7	878	62.1	
	resident	98	1.9	259	18.3	
Complications	yes	406	7.7	124	8.8	0.025
Duration surgery (minutes)	mean ± SD	113±60		120±65		<0.001
Thromboembolism prophylaxis during hospital stay	no thromboembolism prophylaxis	904	17.3	219	15.5	0.449
	thromboembolism prophylaxis	3696	70.5	1040	73.6	
	anticoagulation	147	2.8	52	3.7	
	not specified	493	9.4	103	7.3	
Antibiotics during hospital stay	no antibiotics	90	1.7	20	1.4	<0.001
	prophylactic antibiotics	5057	96.5	1360	96.2	
	antibiotic therapy	93	1.8	34	2.40	
Operation	open reduction, internal fixation	3894	74.3	1055	74.6	<0.001
	closed reduction, internal fixation	860	16.4	273	19.3	
	external fixator	16	0.3	5	0.4	
	prosthesis	470	9.00	81	5.70	
Type of operation	elective	1486	28.4	423	29.9	
	emergency	3754	71.60	991	70.10	

SD: Standard Deviation.

### Predictors for teaching

Our analysis identified several significant predictors for teaching in proximal humerus and humerus shaft fracture surgeries. The type of insurance coverage emerged as a significant factor, with statutory insurance being more prevalent in teaching cases compared to private insurance. In terms of surgical procedures, both CRIF and ORIF were more likely to be teaching cases compared to those involving prosthesis. Additionally, the absence of comorbidities was a significant predictor for teaching surgeries (Table 3).

**Table 3:** Predictors of teaching.

Parameter	Sig.	OR	95 % C.I. for OR	
			Lower	Upper
Insurance status statutory vs. private	<0.001	2.962	2.549	3.441
Closed reduction, internal fixation vs. prosthesis	<0.001	1.998	1.508	2.647
External fixator vs. prosthesis	0.297	1.744	0.613	4.958
Open reduction, internal fixation vs. prosthesis	<0.001	1.740	1.350	2.243
Comorbidities no vs. yes	<0.001	1.453	1.246	1.695
Anticoagulation vs. thromboembolism prophylaxis	0.126	1.299	0.929	1.816
Length of stay preoperative (days)	0.061	1.031	0.999	1.064
No thromboembolism prophylaxis vs. thromboembolism prophylaxis	0.041	0.839	0.709	0.993
Not specified thromboembolism prophylaxis vs. thromboembolism prophylaxis	<0.001	0.664	0.529	0.835

Sig.: Significance, OR: Odds Ratio, C.I.: Confidence Interval.

### Predictors for complications

In total, 8 % of patients suffered at least one complication during hospitalization (7.7 % in the non-teaching and 8.8 % in the teaching group, *p* = 0.223). The predominant recorded complications included pneumonia, nerve lesions and cardiac arrhythmia in both the non-teaching and teaching group*.* Moreover urinary tract infections were more prevalent in the non-teaching group. Teaching status was not a predictor for complications. However, a higher ASA-score, the need for antibiotic therapy, the use of anticoagulation, location of fracture site (shaft vs. upper end of humerus), higher age and longer duration of surgery were significant predictors of complications (Table 4).

**Table 4:** Predictors of complications.

Parameter	Sig.	OR	95 % C.I. for OR	
			Lower	Upper
ASA IV vs. ASA I	<0.001	4.295	2.119	8.704
Antibiotic therapy vs. antibiotic prophylaxis	<0.001	3.646	2.386	5.570
Anticoagulation vs. thromboembolism prophylaxis	0.001	1.948	1.317	2.881
ASA III vs. ASA I	0.001	1.772	1.260	2.492
Fracture of shaft vs. fracture of upper end of humerus	<0.001	1.771	1.427	2.199
Sex male vs. female	0.009	1.320	1.073	1.624
Admission type emergency vs. registered/planned	0.031	1.271	1.023	1.579
No thromboembolism prophylaxis vs. thromboembolism prophylaxis	0.089	1.266	0.965	1.661
Comorbidity yes vs. no	0.082	1.220	0.975	1.525
ASA II vs. ASA I	0.472	1.112	0.833	1.483
Length of stay preoperative (days)	0.022	1.052	1.007	1.098
Not specified thromboembolism prophylaxis vs. thromboembolism prophylaxis	0.908	1.021	0.718	1.452
Age (years)	<0.001	1.013	1.006	1.020
Duration surgery (minutes)	<0.001	1.006	1.005	1.007
No antibiotics vs. antibiotic prophylaxis	0.253	0.551	0.199	1.529

Sig.: Significance, OR: Odds Ratio, C.I.: Confidence Interval, ASA: American Society of Anesthesiologists classification system.

## Discussion

In recent years, there have been significant shifts in surgical training and education, primarily influenced by alterations in the healthcare system, such as case-based reimbursement models and the implementation of working time directives.

The aim of this study was the examination of the impact of surgeons experience on the clinical outcome in patients with proximal humerus and humerus shaft fractures. We specifically investigated the patient characteristics that necessitate teaching interventions, and whether surgeries executed as teaching interventions are associated with a heightened incidence of complications.

In our opinion, this study has several strengths, including the prospective documentation and the large sample size, which allowed us to control for many co-factors. However, our results should be approached with some limitations in mind. Firstly, the de-identified data obtained from the AQC made it impossible to complete or check for missing relevant information. Missing data was added wherever possible using already available information from other data points. Incomplete datasets were excluded from this study. Second, the quality of the obtained data relied on the care of the physician who collected it. Thirdly, there was no information about other patient characteristics, such as details regarding medications. Fourth, the data did not include the level of training of the residents. Lastly, our analysis was limited to examine in-hospital outcomes, excluding the assessment of long-term effects of teaching and associated costs.

Concerning age, sex, ASA type, length of stay and need for intensive care (>1 day) was no difference detected between the teaching and non-teaching group. Regarding the insurance status, we noticed statistically more patients with statutory insurance in the teaching group, which is consistent with findings in previous studies [12]. This may be due to teaching hospitals serving as public care centres, where statutory insured patients are prioritized to ensure a stedy patient flow for training purposes. Additionally hospital policies and patient selection criteria might lead statutory insurance patients to be handled as teaching cases.

Amongst all surgeries performed, the ASA score was nearly equal in both groups. However, the rate of comorbidities was significantly higher in the non-teaching group. This suggests that patients with a higher rate of comorbidities were less likely patients with teaching operation setting. The most likely reason for this is that the teaching surgeon might expect a higher peri‑ or postoperative risk if the operation time would be prolonged when involving the resident in a major role [13]. In our study, the operation time was also significantly longer in the teaching group. CRIF and ORIF were more likely to be teaching cases compared to those involving prosthesis. Reasons for this are most likely to be the technical demanding nature of these operations [16] as well as the known higher rate of complications for trainees known from arthroplasties of other joints. Unwin et al. reported a higher dislocation rate in patients who underwent hip hemiarthroplasties performed by unsupervised trainees using a posterior approach [14]. In another study investigating hip arthroplasties, training operations had statistically higher rate of revisions [15]. In contrast, Mahaluxmivala et al. [16] investigated 673 cases of total knee arthroplasties and saw no statistical differences concerning the measured alignment of the prosthesis's performed by consultants compared to residents. Concerning shoulder arthroplasty there are some studies investigating primary procedures and their complication rates in correlation with the surgeon experience. However, most of them focus on a single surgeon`s learning curve or the performed volume of the hospital [[17], [18], [19], [20]]. Yet, Sabesan et al. [21] found no increase in complications in teaching hospitals compared to non-teaching hospitals investigating 24,056 discharges for patients undergoing reverse shoulder arthroplasty. Due to these inconclusive results among the literature concerning complications as well as the high demanding procedure, fracture arthroplasty of the shoulder seem to be rather a contradictor for a teaching procedure.

Our results showed no statistical difference concerning the rate of complications during the hospital stay between teaching operations and non-teaching operations. A review investigating appendectomies discovered no statistical association between teaching operations and complications or mortality. Nevertheless, a longer duration of surgery was shown in the teaching group in comparison to the non-teaching group [22]. In contrast, Matulewicz et al. [23] showed in a study involving 40,000 urological cases no enhanced rate of complications.

In the case of orthopedic and trauma surgery there was no significant difference reported concerning early complication rate following proximal femoral nailing or hemiarthroplasty for femoral neck fractures [[24], [25], [26]]. In 2009, Palan et al. reported in a multicentre prospective study no significant differences for total hip replacements amongst 1501 patients between trainers and trainees as well as operations where the surgeons assisted or the trainee was the assistant [27]. Pugely et al. evaluated 8221 lower extremity traumas, 4750 spine arthrodesis, 16,832 basic and 5916 advanced arthroscopies as well as 28,686 primary total joint arthroplasties and 2412 revision joint arthroplasties concerning short term major morbidity as well as mortality due to involvement of a resident and found no statistical differences [28].

To our knowledge there is only one study reporting complication rate of residents amongst humeral fracture treatment [11]. Altinas et al. showed in 1134 cases of proximal humeral nailing that there is no difference in major in-hospital complication comparing operation performed by attending or residents. Our study showed consistent results, despite concerning the mortality rate. Here we found a slightly decreased rate among the group of teaching operations. However, it was not statistically different. This could be caused by the selection of patients who qualify for a teaching operation.

Despite the increasing availability and usage of virtual reality and box model simulations [[29], [30]] the training in the operation room through assistance of a senior surgeon resides to be main column of surgical learning procedures [31].

Resident training should be considered an ethical and safe method of learning, provided that supervised resident participation does not have too great an impact on postoperative outcomes concerning the surgical treatment of humeral fractures [32].

## Conclusion

The teaching of surgical procedures showed no impact on in-hospital mortality among patients with a shaft or proximal humeral fracture. However, teaching was an independent predictor of a prolonged duration of surgery. Despite a lower incidence of concomitant comorbidities in the teaching group, there was no significant difference in their in-hospital outcome. Nevertheless, the differences seem to be insignificant when considering the benefits of teaching. In future studies, the long-term outcomes of teaching surgeries could be explored on the impact of resident participation on patient satisfaction and cost-effectiveness.

## Funding

This research received no external funding.

### Ethical review committee statement

The data of this study are based on anonymized, de-identified data; our institutional review board waves the necessity of institutional review board approval.

### Statement of location where the work was performed

Division of Trauma Surgery, University Hospital Zurich (USZ), University of Zurich (UZH), Raemistrasse 100, 8091 Zuerich, Switzerland

### Ethical review committee statement

The data of this study are based on anonymized, de-identified data; our institutional review board waves the necessity of institutional review board approval.

## CRediT authorship contribution statement

**Andrew Adams:** Writing – review & editing, Writing – original draft, Methodology, Formal analysis, Data curation. **Christina Lorenz:** Supervision. **Valentin Neuhaus:** Visualization, Validation, Supervision. **Hans-Christoph Pape:** Validation, Supervision, Conceptualization. **Claudio Canal:** Supervision.

## Declaration of competing interest

The authors declare that they have no known competing financial interests or personal relationships that could have appeared to influence the work reported in this paper.

## Appendix Supplementary materials

Image, application 1
